# Understanding the public’s profile of mental health literacy in China: a nationwide study

**DOI:** 10.1186/s12888-018-1980-8

**Published:** 2019-01-14

**Authors:** Debbie Huang, Lawrence H. Yang, Bernice A. Pescosolido

**Affiliations:** 1 Mailman School of Public Health, Columbia University, 722 W. 168th St., New York, NY 10032 USA; 2 New York University College of Global Public Health, 715 Broadway, Room 1212, New York, NY 10003 USA; 3 Department of Sociology, Indiana University, 1022 E Third St, Bloomington, IN 47405 USA

**Keywords:** Global mental health, Mental health literacy, Mental health recognition, Psychiatric epidemiology, Mental health service-use

## Abstract

**Background:**

In the wake of China’s massive economic development, attention has only recently turned to the enormous treatment gap that exists for mental health problems. Our study is the first comprehensive, national examination of the levels and correlates of the public’s ability to recognize mental illness in the community and suggest sources of help, setting a baseline to assess contemporary Chinese efforts.

**Methods:**

Data were collected in China as part of the Stigma in Global Context – Mental Health Study (SGC-MHS) through face-to-face interviews using vignettes meeting clinical criteria for schizophrenia and major depression. Our analysis targets the Han Chinese participants (*n* = 1812). Differences in the recognition of mental health problems were assessed using a chi-square test and further stratified by vignette illness type and urban vs. rural residence. Adjusted regression models estimated the effects of each predictor towards the endorsement three types of help-seeking: medical doctor, psychiatrist, and mental health professional.

**Results:**

As expected, recognition of mental health problems is low; it is better for depression and most accurate in urban areas. Perceived severity increases endorsement of the need for care and for treatment by all provider types. Recognition of a mental health problem specifically decreases endorsement of medical doctors while increasing recommendations for psychiatrists and mental health professionals. Neurobiological attributions decrease recommendations for mental health professionals as opposed to general or specialty physicians.

**Conclusions:**

Continued efforts are needed in China to promote mental illness recognition within rural areas, and of schizophrenia specifically. Promoting recognition of mental illness, while balancing the special challenges among individuals who understand the neurobiological roots of mental illness, may constitute a key strategy to reduce the sizeable mental health treatment gap in China.

## Background

In the wake of China’s massive economic development, attention has turned to the enormous treatment gap that exists for mental health problems. Phillips et al.’s foundational study [1], representing 12% of China’s population, assessed the prevalence of any mental disorder at 17.5%, with estimates indicating that more than 91% of 173 million Chinese citizens with a mental disorder fail to reach professional help [2]. In 2011, China passed its first National Mental Health Law, articulating ambitious reform for the mental health service delivery system [3, 4].

Implementing this mental health reform is challenging as closing the mental health gap requires a multi-level approach, targeting both the service structure and individual-level factors. To expand mental health coverage for both psychotic (e.g., schizophrenia) and especially non-psychotic disorders (e.g., depression), the new policy aims to increase the voluntary use of mental health services by improving treatment accessibility outside the tertiary sector [1, 4]. Yet, poor mental health literacy may prevent or deter individuals from making use of appropriate treatment options. The term mental health literacy is defined as “knowledge and beliefs about mental disorders which aid their recognition, management or prevention [5].” As such, literacy goes beyond recognition to include knowledge about help-seeking and proper interaction with providers, all of which can increase the rate of detection and treatment [6, 7] . Accordingly, having a rigorous, representative profile of the population’s basic mental health literacy can help in ascertaining the general public’s capacity to make effective use of the planned expansion of mental health services, as well as to allow policymakers to plan for targeted improvement of mental health recognition among groups where literacy is low.

Existing studies of mental health literacy in China have suggested that individuals with better recognition of mental disorders have greater endorsement of seeking help from mental health professionals [8, 9]. Among those with lower mental health recognition, mental illness is categorized as a ‘personal deficit’ which translates to lower mental health utilization and adoption of individual coping strategies that delay utilization of any medical treatment [10, 11]. Yet seeking general medical care is often insufficient for appropriate psychiatric care. For example, among those who first sought help in a general medical (i.e., non-psychiatric) facility, fewer than 10% of individuals received an accurate psychiatric diagnosis and timely referral to psychiatric treatment [12]. Rather, accurate recognition of mental illness has been seen to improve specific recommendations for help-seeking from mental health professionals in China [8, 9]. Further, among mental disorders, the recognition of schizophrenia has been shown to be lower compared with other disorders such as depression (in rural Hunan, urban Beijing, urban Hong Kong, and Shanghai) [8, 11–13]. Finally, research in the Chinese context has also found some socio-demographic influences. Older individuals tend to have lower levels of mental illness recognition [11, 14]. Despite high rates of mental illness among females, there appear to be fewer gender differences in levels of recognition of depression or schizophrenia [11, 15].

Although these prior studies suggest that improving recognition is an important issue in China, these studies have several key limitations. Many studies use small samples; are restricted to particular geographic regions (e.g. rural Hunan, urban Beijing, urban Hong Kong, or rural Hunan and Shanghai); or only target specific subgroups (e.g. caregivers or non-mental health healthcare professionals); and underplay the role of socio-demograhics like age and gender [8, 11, 16, 17]. Given that there has yet to be a large-scale national study of mental illness literacy in China, the Stigma in Global Context – Mental Health Study offers data to fill this gap. Specifically, we first aim to provide descriptive data on two basic aspects of mental health literacy — problem recognition and treatment recognition among the Chinese population in response to vignettes describing (but not identifying) hypothetical individuals meeting diagnostic criteria for depression and schizophrenia. Second, we provide multivariate analyses of the vignette and socio-demographic correlates of mental health literacy.

From a population health perspective, promoting accurate recognition of mental illness to facilitate contact with practitioners with mental health training would be a useful step in addressing China’s mental health gap. Using data from a national sample of residents in China from the Stigma in Global Context – Mental Health Study (SGC-MHS), we anticipate that 1) there are likely differences in the recognition of different mental health problems (i.e., schizophrenia vs. depression); and, 2) the accurate recognition of problems as mental illness will be associated with help-seeking recommendations from medical sources (i.e., seeking help from a general medical doctor, psychiatrist, or mental health professional), even after accounting for other known influences, including perceived severity of the condition and to what extent the condition is perceived as having a neurobiological cause [18–20].

## Methods

### Sample

Data from the Chinese population was originally collected as part of the Stigma in Global Context-Mental Health Study (SGC-MHS), whose goal was to understand the level of stigma in 16 countries in the Global North and Global South [21]. Participants were selected based on a cross-sectional multistage probability sampling method to select a nationally-representative adult sample from all geographical regions of Mainland China. Specifically, a four-stage cluster stratified random sample design with unequal probabilities of selection of China’s 2803 county or district-level administrative units was conducted within 22 provinces, 4 autonomous regions, and 4 municipalities (details available on request). Eligible respondents were non-institutionalized adults (i.e., 18 years of age or older). Data were collected were conducted by trained staff who were closely monitored by survey center personnel at Renmin University in Beijing as part of the Chinese General Social Survey. Professor Weidong Wang served as a liaison on translation, data coding, and preparation and delivery of data files. In China, interviewers were recruited in local regions to accommodate variations in Chinese dialects. IRB approval for the SGC-MHS, as a whole, is held at Indiana University (Study #04–9051).

**Fig. 1:**
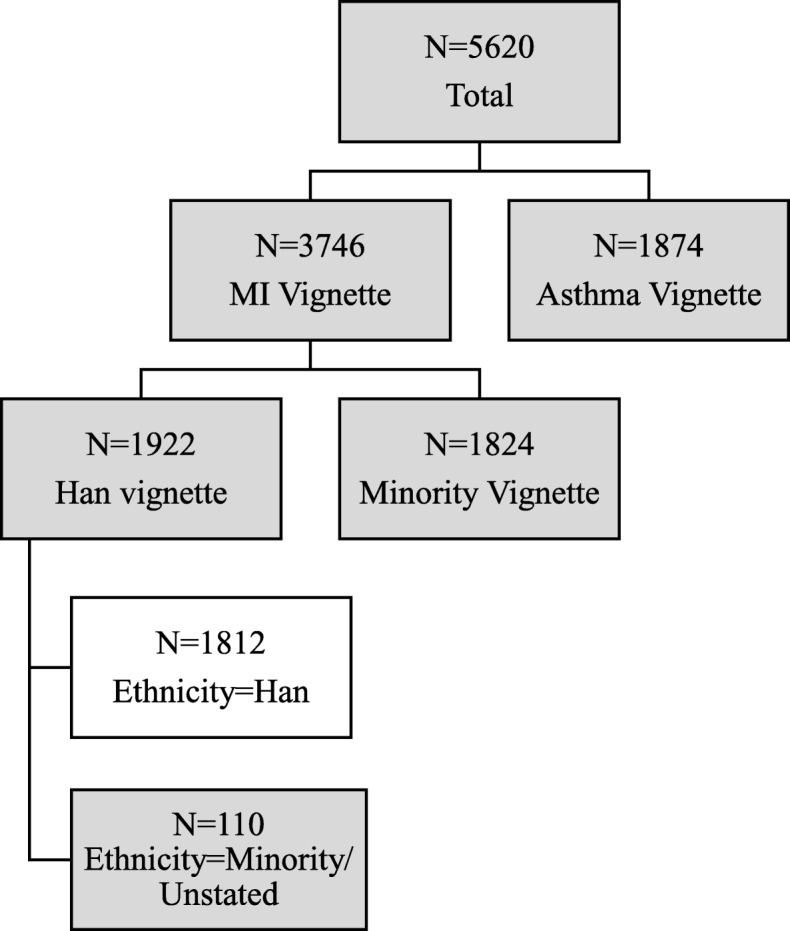
Sample Characteristic Flow Chart, Han Chinese Component of the Stigma in Global Context – Mental Health Study (SGC-MHS), 2011

A total of 5617 Chinese respondents participated. In China, ethnic minorities typically reside in designated regions with preferential policies for education, economic development [22], and health care services [23]. Such substantial systematic differences are likely to affect mental health literacy, including recognition, which is the focus of this study. To remove these complexities stemming from systematic and structural differences, this study focuses on characterizing the profile and processes underlying mental health recognition of the majority (Han) ethnic group in China (Effective sample size = 1812; see Fig. 1). The Han ethnic group constitutes approximately 91.5% (2010) of the total Chinese population. Therefore, results from this sample are generalizable to a sizeable proportion of the Chinese population. Based on the flow diagram, 94.4% of our sample is Han. Table 1 provides basic demographics with our sample demonstrating broad alignment with census population profiles. However, typical differences from population statistics in survey research (e.g., a slight over-representation of women) were also in evidence [24].

**Table 1:** Sample and census characteristics, Han Chinese component of the SGC-MHS, 2011 (*N* = 1812)

	Mean or Frequency %	2010 China Census %
Age in Years^a^		
< 35 years old	24.28	16.1 (0–14 years old)
36–64	59.93	70.3 (15–60 years old)
65+	15.78	13.6 (61+)
Mean (SD)	47.40 (16.24)	
Gender		
Male	43.6	51.2
Female	56.4	48.8
Income in RMB- Mean (SD)		
Personal	1435.26 (4406.60)	
Household	9489.73 (248915.49)	
Marital Status		
Married/Cohabited	86.9	92
Widowed/Divorced/Separated	3.0	
Single	10.1	
Education Attainment^a^ (Includes ages 6+)		
No Schooling	9.4	4.7
Primary School	18.8	27.8
Junior High School	25.4	42.3
High School Graduate/Technical School	17.3	21.1
Higher Education (University)	12.5	4.1
Missing	16.6	
Residence		
Urban	31.7	31.6
Rural	68.3	68.4

Note: Census characteristics for variables in asterisk^a^ include populations under 18 years old; our study only includes individuals 18 years and older. *N* number of subjects, *%* percentage, and *SD* standard deviation

The SGC-MHS instrument consisted of two sections. The first section includes 75 items that tapped into substantive issues related to the recognition, understanding, treatment suggestions and attitudes via reference to a specific case (described below) and more generally with regard to mental illness. Most items in the core interview had been used in previous research, many from extant scales with known psychometric properties. The second part of the interview schedule consisted of an agreed-upon set of 14 socio-demographic background variables that have been tailored to each nation by ISSP teams (see the Zentralarchiv, the ISSP designated archive in Germany).

A vignette strategy, where cases are not identified as mental illness, allow for data collection on knowledge and recognition among respondents [25]. These ethnographically-grounded vignettes were developed to present symptoms and behaviors of hypothetical persons with two major mental illnesses, schizophrenia and major depression according to Diagnostic & Statistical Manual (DSM-IV) criteria (physical health vignette of asthma not used here) [26]. All vignettes were evaluated for accuracy by a psychiatrist with expertise in cross-national studies of psychiatric disorders, for acceptability by the research team at Renmin University, and for cultural understanding by Chinese native speakers (see Pescosolido et al. for more detail) [27] [26]. Each vignette was constructed to vary by gender, education, and the majority/minority race/ethnicity. Respondents received one randomly assigned vignette. Because all respondents in these analyses were of Han ethnicity, only responses towards Han vignette individuals are used here. Vignettes are provided in Appendix 1.

### Measures

#### Mental illness recognition

[NAME] is used here as a generic term to refer to the vignette person; in the survey, typical male and female Han names were used. Participants were asked, “Would you say that [NAME’s] situation is caused by depression, asthma, schizophrenia, stress, or something else?” Exact/specific mental illness recognition was defined as correctly identifying the vignette character’s specific mental illness diagnosis (i.e., depression or schizophrenia). This variable was recoded from the original response categories into a binary variable (correct/ incorrect). We also assessed “general” mental illness recognition, which was defined as identifying some type of mental illness diagnosis, even if the specific diagnosis was incorrect (e.g., identifying the vignette as depression when it depicted schizophrenia). This variable was also dichotomized.

#### Treatment recommendations

The endorsement or recommendation for three types of formal help-seeking outcomes, “a general medical doctor,” “a psychiatrist,” and “mental health professional,” were assessed. Participants were asked, “For each statement, please tell me using a scale from 1 to 10, where 1 is not at all important and 10 is very important, how important you think each would be for NAME to use in order to get help with his/her situation.” Mental health professionals include psychologists, therapists, social workers, or counselors; examples were provided if respondents asked for clarification. In a fourth response, respondents were asked, “How likely is it that [NAME’s] situation will improve on its own?” The original response set, a 4-point Likert likelihood scale, was recoded into a binary outcome (likely/not likely).

#### Biological/genetic causal belief

Respondents were asked the likelihood that “[NAME’s] situation” was caused by a “brain disease or disorder” or a “genetic or inherited problem.” Endorsing either one of these attributions was coded as a neurobiological binary variable (likely/not likely).

#### Perceived illness severity

Respondents were asked, “How serious would you consider [NAME’s] situation to be?”. Participants responded using a 4-point Likert scale of “very serious, moderately serious, not very serious, to not at all serious”, which was reverse-coded so that a higher score indicates higher severity.

#### Sociodemographic control variables

Age was coded in years. Gender was dichotomized (male = 1) as was rural/urban residence (urban = 1). Income was measured as a continuous variable. Marital status and educational attainment were originally included as dummy variable sets in the preliminary analysis (categories listed in Table 1). However, because of their high inter-correlation with rural/urban status, they were dropped from final analyses.

### Analysis

Simple descriptive data, univariate tests and multivariate analyses are used to provide the profile of recognition and response options and to examine their correlates. Specifically, differences in the percentage of respondents who accurately recognize the general and specific disorder described in the vignette is assessed by vignette illness type using a chi-square test. Results are further stratified by urban versus rural residence. Bivariate analysis between social demographics and outcome variables using ANOVA and Pearson Chi-square Test were also conducted to identify key sociodemographic factors to be included in the model. Given that education, income, and marital status were highly related to rural/urban residence in both the literature and in our analysis, the final model included only rural/urban residence. Multiple series of adjusted regression models estimated the effects of each predictor towards the three types of help-seeking. Ordinary least square (OLS) and logistic regression modeling were used depending on whether the outcome was dichotomous or continuous. The four steps look at independent effects of key social demographic factors (Model 1), with accurate mental illness recognition added (Model 2), considering biological/genetic causal beliefs (Model 3), and finally adding perceived illness severity (Model 4). Lastly, because age suggested a nonlinear relationship with mental health recognition in prior studies [11, 14] we explored interaction effects between: a) age groups (defined categorically as ≤35 years old, 36–64 years old, and ≥ 65 years old, with 36–64 years acting as the reference category because this group might be expected to have the greatest recognition of mental illness [11, 14]); and b) gender, to determine whether the effects of recognition varied (i.e., were modified) by these two factors (Model 5 and 6). These models were run both for specific and general mental illness recognition. Statistical significance was set at *p* < 0.05. All analysis was conducted using Statistical Package for Social Sciences (SPSS) Version 22.

## Results

### Levels of accurate mental illness recognition

Table 2, Panels A-B reports the percentage of Chinese Han respondents who could recognize the vignettes as problems of mental illness (Panel A) and, more specifically, as the DSM disorder described (Panel B). Three findings stand out. First, the rates of recognition are low but substantial. Just under one-third of respondents (32.6%) were able to generally recognize the vignette as a mental illness. Second, only 18.6% were able to accurately recognize the specific mental illness depicted. When stratified by urban versus rural residence, a significantly greater percentage of urban respondents (23.3%) were able to accurately recognize the exact diagnosis in the vignette compared to rural residents (16.4%). Third, across mental illness types, accurate recognition of depression (25.5%) was significantly greater than accurate recognition of schizophrenia (11.2%). Among urban respondents, this difference was even greater; 38.0% of respondents correctly identified depression while only 8.7% correctly identified schizophrenia (*p* < 0.001). In contrast, among rural respondents, correct recognition of depression (20.0%) and schizophrenia (12.4%) did not significantly differ.

**Table 2:** Correct recognition and treatment recommendations of vignette overall and by urban and rural residence

Recognition - Vignette Type			
	Urban %	Rural %	All % (*N*)
Panel A: General Recognition (Mental Illness)			
For Both Disorders (*N* = 1812)	39.7^a^	29.2^a^	32.6 (590)^a^
For Schizophrenia Vignette (*N* = 789)	32.1	25.9	27.9 (244)
For Depression Vignette (*N* = 548)	47.4^a^	32.3^a^	36.9 (346)^a^
Panel B: Exact Recognition			
For Both Disorders (*N* = 1812)	23.3^a^	16.4^a^	18.6 (337)^a^
For Schizophrenia Vignette (*N* = 789)	8.7	12.4	11.2 (98)
For Depression Vignette (*N* = 548)	38.0^a^	20.0^a^	25.5 (239)^a^
Panel C: Will Not Improve on Its Own			
For Both Disorders (*N* = 1712)	41.2^a^	34.2^a^	36.4 (624)^a^
For Schizophrenia Vignette (*N* = 830)	39.9	34.2	36.1 (300)
For Depression Vignette (*N* = 882)	42.6^a^	34.2^a^	36.7 (324)^a^
	Urban $\overline{x}$ (SD)	Rural $\overline{x}$ (SD)	All $\overline{x}$ (SD)
Panel D: Recommend Medical Doctor			
For Both Disorders (*N* = 1777)	6.41 (2.75)	6.44 (2.77)	6.43 (2.76)
For Schizophrenia Vignette (*N* = 856)	6.61 (2.77)	6.90 (2.63)	6.80 (2.68)
For Depression Vignette (*N* = 921)	6.22 (2.71)	6.02 (2.82)	6.08 (2.79)
Panel E: Recommend Mental Health Professional			
For Both Disorders (*N* = 1758)	7.91 (2.59)^a^	7.41 (2.71)^a^	7.57 (2.68)^a^
For Schizophrenia Vignette (*N* = 854)	7.33 (2.88)^a^	6.84 (3.04)^a^	7.00 (3.00)^a^
For Depression Vignette (*N* = 904)	8.51 (2.09)^a^	7.93 (2.25)^a^	8.11 (2.22)^a^
Panel F: Recommend Psychiatrist			
For Both Disorders (*N* = 1742)	6.13 (3.29)	6.07 (3.24)	6.09 (3.26)
For Schizophrenia Vignette (*N* = 840)	5.57 (3.42)	5.57 (3.41)	5.57 (3.41)
For Depression Vignette (*N* = 902)	6.68 (3.05)	6.53 (3.02)	6.58 (3.03)

Note: *N* number of subjects, *%* percentage, $\overline{\boldsymbol{x}}$= mean, and *SD* standard deviation; ^a^Chi-square Test/ANOVA, *p* < 0.05

### Levels of treatment recommendations

Table 2, Panels C-F reports the public’s assessment of appropriate response to the vignette situation. Just over 36% indicated that the situation will ***not*** improve on its own. In terms of the importance of medical professionals, the population average for mental health professionals was highest (Mean = 7.57, SD = 2.68), followed by medical doctors (Mean = 6.43, SD =2.76), and lastly, by psychiatrists (Mean = 6.09, SD = 3.26).

### Correlates of recognition and recommendations

Table 3 presents the multivariate results. All models were run with both exact and general mental illness recognition, but only the former are presented. Preliminary results indicated almost no difference in significance and no meaningful differences in magnitude between the recognition variable types nor significant change in the effects of other correlates, though differences will be noted.

**Table 3:** Adjusted logistic regression analysis of treatment recommendations

Panel A: Situation Will Not Improve On Its Own (*N* = 1620)				OR (95% CI)								
				Model 1		Model 2			Model 3		Model 4	
Age (<=35 vs. 36–64)				0.91 (0.72, 1.16)		0.91 (0.71, 1.16)			0.94 (0.73, 1.2)		0.93 (0.73, 1.2)	
Age (> = 65 vs. 36–64)				1.13 (0.84, 1.51)		1.12 (0.83, 1.5)			1.09 (0.81, 1.47)		1.1 (0.82, 1.48)	
Male (1)				1.05 (0.85, 1.29)		1.05 (0.85, 1.29)			1.06 (0.86, 1.3)		1.06 (0.86, 1.3)	
Urban (1)				1.33 (1.07, 1.65)*		1.28 (1.03, 1.59)*			1.29 (1.04, 1.6)*		1.32 (1.06, 1.64)*	
Exact Recognition+ (1)						1.81 (1.41, 2.32)*			1.82 (1.42, 2.34)*		1.71 (1.33, 2.21)*	
Illness Explanation-Biological/Genetic Cause (1)									1.26 (1.01, 1.57)*		1.22 (0.97, 1.52)	
Perceived Illness Severity											1.36 (1.17, 1.58)*	
Panel B: Importance of Specific Providers												
Variable	Importance of Medical Doctor				Importance of Psychiatrist				Importance of Mental Health Professional			
	ß (95% CI)^a^				ß (95% CI)^b^				ß (95% CI)^c^			
	Model 1	Model 2	Model 3	Model 4	Model 1	Model 2	Model 3	Model 4	Model 1	Model 2	Model 3	Model 4
Age (<=35 vs. 36–64)	−0.45 (−0.76, −0.14)*	− 0.44 (− 0.76, − 0.13)*	− 0.34 (− 0.65, − 0.03)*	− 0.34 (− 0.65, − 0.03) *	− 0.45 (− 0.82, − 0.09)*	− 0.47 (− 0.83, − 0.1) *	−0.39 (− 0.76, − 0.02) *	−0.4 (− 0.77, − 0.04) *	0.02 (− 0.28, 0.32)	0.01 (− 0.28, 0.31)	−0.04 (− 0.34, 0.25)	−0.05 (− 0.34, 0.24)
Age (> = 65 vs. 36–64)	0.3 (− 0.09, 0.68)	0.31 (− 0.07, 0.69)	0.24 (− 0.14, 0.62)	0.24 (− 0.14, 0.62)	0.3 (− 0.16, 0.75)	0.27 (− 0.18, 0.72)	0.21 (−0.24, 0.66)	0.25 (− 0.2, 0.69)	−0.48 (− 0.85, − 0.1)*	−0.51 (− 0.88, − 0.14) *	−0.47 (− 0.84, − 0.1) *	−0.42 (− 0.78, − 0.06) *
Male (1)	−0.27 (− 0.54, − 0.01)*	−0.27 (− 0.53, 0)*	−0.24 (− 0.5, 0.02)	−0.24 (− 0.5, 0.02)	0.08 (− 0.23, 0.4)	0.08 (− 0.23, 0.39)	0.1 (− 0.21, 0.41)	0.1 (− 0.2, 0.41)	0.06 (− 0.2, 0.31)	0.05 (−0.2, 0.31)	0.04 (− 0.21, 0.29)	0.04 (− 0.2, 0.29)
Urban (1)	0.01 (− 0.27, 0.29)	0.06 (− 0.22, 0.34)	0.07 (− 0.21, 0.35)	0.07 (− 0.21, 0.35)	0.07 (− 0.27, 0.4)	−0.02 (− 0.35, 0.32)	−0.01 (− 0.34, 0.33)	0.03 (− 0.3, 0.36)	0.49 (0.21, 0.76)*	0.4 (0.13, 0.67) *	0.39 (0.12, 0.66) *	0.42 (0.16, 0.69) *
Exact Recognition+ (1)		−0.66 (− 1, − 0.33)*	−0.65 (− 0.98, − 0.31) *	−0.65 (− 0.99, − 0.32) *		1.14 (0.75, 1.53) *	1.15 (0.76, 1.54) *	0.99 (0.6, 1.38) *		1.24 (0.92, 1.55) *	1.23 (0.91, 1.55) *	1.07 (0.76, 1.39) *
Illness Explanation-Biological/Genetic Cause (1)			0.75 (0.48, 1.03) *	0.75 (0.47, 1.03) *			0.54 (0.21, 0.87) *	0.45 (0.12, 0.78) *			−0.39 (−0.65, − 0.12) *	−0.47 (− 0.74, − 0.21) *
Perceived Illness Severity				0.02 (−0.17, 0.21)				0.71 (0.49, 0.93) *				0.68 (0.51, 0.86) *

Note. *N* number of subjects, *CI* Confidence interval, *OR* odds ratio, *ß* beta coefficient; *p < 0.05

^+^Model using some MI recognition were conducted and no difference between exact and some recognition were observed

^a^Population size (N) = 1671, R^2^ = 0.041; ^b^ Population size (N) = 1649, R^2^ = 0.056; ^c^ Population size (*N*) = 1655, R^2^ = 0.081

Panel A considers the correlates of individuals who believe that the vignette condition will not improve on its own. In Model 1, those living in urban areas, compared to those living in rural areas, report that the condition described in the vignette would not get better on its own. Adding the main effects of exact recognition does not change the sociodemographic effects, and exact recognition was significantly associated with believing that the vignette condition would not get better on its own. The next two steps, endorsing neurobiological causes for the vignette situation and perceived severity, respectively, increased the likelihood of seeing the vignette condition as not getting better on its own, with only perceived severity emerging as a significant correlate in Model 4. The effects of exact recognition were not modified by gender nor age categories (with the middle age category of 36–64 years acting as the reference category; results not shown for interactions for gender or age). In general, the results did not differ when exact recognition was substituted with general recognition.

Panel B examines the correlates of respondents’ assessment of the importance of different providers. In this case, younger individuals (≤35 years old) are less likely to see medical doctors and psychiatrists as important. However, this age effect is reversed for older individuals who are significantly less likely to recommend mental health professionals. Exact recognition is significantly associated with lower recommendations for medical doctors; and, it increases the public’s endorsement of specialty providers (both psychiatrists and mental health professionals; results similar in magnitude and significance for general recognition). Endorsement of biological or genetic attributions is significantly associated with greater importance of medical doctors and psychiatrists but with less importance of mental health professionals. Across the board, severity increases the recommendations of psychiatrists and mental health professionals.

**Fig. 2:**
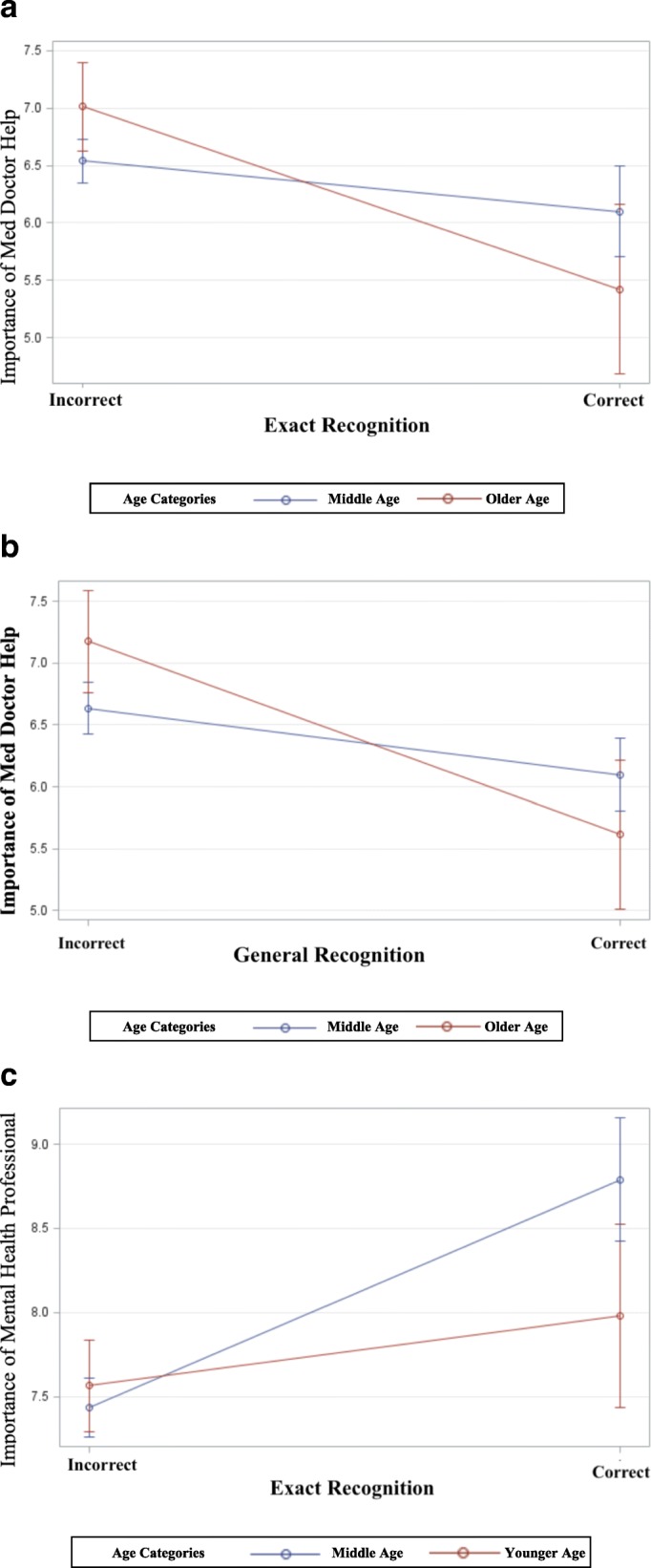
**a** Exact Recognition and Importance of Medical Doctor (Older versus Middle Age). **b** General Recognition and Importance of Medical Doctor (Older versus Middle Age). **c** Exact Recognition and Importance of Mental Health Professional (Younger versus Middle Age)

The effects of exact recognition on respondents’ assessment of the importance of different providers were not modified by gender. When examining interactions between exact recognition and age, the effects of exact recognition were modified by age group for recommendations for medical doctor and for mental health professionals. The relationship between exact recognition and lower recommendation for medical doctor is stronger for older individuals (≥65 years old) than middle adults (36–64 years old; see Fig. 2a). The relationship between exact recognition and higher recommendation for mental health professional is weaker for younger people (≤35 years old) than middle age individuals (36–64 years old; see Fig. 2c). Interaction results remained consistent for age group and recommendation for medical doctor when substituting general recognition for exact recognition (see Fig. 2b) but not for recommendation for mental health professional (results not shown).

## Discussion

### Mental health recognition in China

Overall, the recognition of mental illness can be characterized as relatively low in our sample. This may be expected, as is the lower recognition of schizophrenia relative to depression, a finding that remained consistent after stratifying by geographic residence. This level of recognition is much lower than in Western nations (Switzerland, Germany, and Australia) [28–30], where depression awareness in nationally-representative studies is between 37.5–73.7% and schizophrenia is 22.4–73.6%. However, this is consistent with other recognition findings within China (schizophrenia = 8.5–10.0%; depression = 16.1–36.3%). The results of this study suggest continued efforts are still needed, particular to address the gap in mental health recognition between urban and rural residents. The dedicated efforts by China’s 2002–2010 Mental Health Work Plan to increase public recognition of common mental disorders, specifically depression and dementia, might partially account for the greater observed accurate recognition of depression in our study.

Prior explanations of relatively poor mental health recognition identify several key contributory factors, including lack of public mental health education and awareness [11], culturally-specific idioms of mental illness that provide a socially-accepted interpretation of symptoms (i.e., “excessive thinking”) [31]; and severe stigma of mental illness [32]. The negative stigma towards mental illness among Chinese populations may have contributed to a reluctance to associate symptoms with mental illness and to label the individual as having a mental disorder [33]. Instead, there is a greater tendency to attribute socially or culturally appropriate labels (e.g., work problems or stress) to mental illness symptoms [3, 13, 34]. As the first study, to our knowledge, to utilize a nationally-based sample to capture baseline mental health literacy and its influence on different treatment recommendations for China, our findings complement the emergence of China’s 2002–2010 Mental Health Work Plan [35]. Adopting a purely diagnostically-based approach, the plan aims to improve public awareness of the characteristics of mental illnesses in order to build both help-seeking and advocacy in Chinese society. Other evaluations of this plan have not been confined to Western vignettes of psychiatric disorders. Rather, some have evaluated mental health literacy through respondent’s understanding of multiple facets of mental health (i.e., assessing both “mental disorder” and “mental health” literacy via the 20-item Mental Health Knowledge Questionnaire [MHKQ]). These broader measures of mental health literacy do indeed result in relatively higher levels of mental health literacy, as defined in a multi-component manner among both rural (58%, [14]) and urban (60–72%; [36–39]) Chinese community respondents. We believe that both approaches—a diagnostically-focused one that assesses recognition and response to psychiatric syndromes, and a broader strategy incorporating awareness of features and prevention of mental disorders—hold utility in improving recognition of specific mental illnesses as well as improving broader knowledge and promotion of mental health and mental disorders.

### Core components of treatment recommendations

The SGC-MHS also allowed for an analysis of how different aspects of mental illness recognition and beliefs are associated with endorsement of formal treatment options. The ability to recognize a scenario as a mental health problem, whether generally or specifically, consistently emerged as a significant predictive factor even while controlling for other demographic and key attitudinal factors. Respondents who were able to accurately recognize the vignette tended to see a greater importance in seeking help from psychiatrists and mental health professionals. This extends research where respondents who did not see mental illness as a personal failure, but rather as a disorder, were more like to endorse treatment [8, 19, 40].

The prominent somatization of mental illness symptoms, made popular during the Cultural Revolution [34, 41], may have influenced why some respondents attributed the cause of the vignette to be biological/genetic in nature and why physical/genetic causal belief was a significant predictor in all of the treatment recommendations [8, 20]. As in many mental health services research studies, greater perceived severity was associated with treatment recommendations for psychiatrists and/or mental health professionals [42]. But the differential response to mental health professionals among some variables suggests that special effort should be targeted to their acceptance, especially as more of these providers are trained to help address China’s mental health gap. As currently stated in the 2015–2020 National Mental Health Work Plan [43], improving the lay public’s attitude towards, and understanding of when to utilize, specialized mental health providers, is not specifically addressed.

We did not find any gender differences in the relationship between recognition and treatment recommendation. Gender effects on treatment recommendation have not been previously examined in China. One study [11] did demonstrate that females were more likely to recognize alcohol abuse. However, several other studies in China [11, 15, 36] found that gender was not associated with accurate recognition of schizophrenia or depression. These findings contrast with studies conducted in Western contexts where females generally tend to show better mental health recognition [44, 45]. One potential explanation may be a floor effect in China; since the recognition of schizophrenia and depression is very low overall, gender-related effects upon recognition may be less detectable.

Our study did find age-specific differences. Prior studies in Western contexts and China have suggested that older age was associated with lower mental health literacy [11]. However, our findings suggest that relationships with age and recognition of mental illness are more nuanced in regards to specific forms of mental health treatment in China. There is a greater tendency to see the importance of seeking out a mental health professional when a middle-aged person (i.e. ages 36–64 years old) can recognize any form of mental illness. This may reflect greater life exposure to mental illnesses and health care, when compared with a younger person (≤35 years old), who may lack such experiences [46]. Counterintuitively, our findings also showed that older individuals (≥65 years old) showed a greater tendency, when recognizing either the specific or general form of mental illness, to not recommend a medical doctor for treatment, compared with a middle-aged person (i.e. ages 36–64 years old). It is possible that these older groups may not subscribe to general medical treatment when recognizing mental illness, opting instead for informal network support (e.g., from family), or other alternative (e.g., Chinese herbal medicine) forms of treatment [13]. Regardless, our results demonstrate that the relationship of mental illness recognition, age, and treatment recommendations is not linear in China. Further, older populations, in particular, might benefit from education campaigns encouraging treatment-seeking from mental health professionals when mental illness is recognized.

### Limitations

The SGC-MHS is cross-sectional thus limiting causal inference. We also acknowledge the possibility that Chinese participants responded to vignettes according to their culturally-informed model of psychiatric distress (e.g., stress). This fits the cultural context, but does not match a traditional Western psychiatric diagnosis. However, since mental health service use in the Chinese medical system is delivered based upon an international psychiatric diagnostic system (ICD 10) [47], we do not view this as impacting the validity of our findings. Further, respondent burden limited the ability of the SGC-MHS to more deeply investigate cultural beliefs or social influence processes linked to networks [42]. Finally, this is an analysis of recommendations for others. Recommendations might be substantially different from what individuals would do when actually confronted themselves with the situation described since lack of an attitude-behavior linkage has been well-documented. We conceptualize this study as a description of the contours of the “cultural toolbox” of beliefs, attitudes, and cultural scripts among the Han Chinese.

These issues should be the focus of future study in China and elsewhere because they are problem generic to much mental health utilization research. In particular, given that the National Mental Health Law (2012) and subsequent reform took place just after the 2011 date of the SGC-MHS, there needs to be a follow-up to document possible shifts in contemporary recognition of mental illness and public support for treatment options. In essence, our study provides a valuable baseline by which to characterize mental health recognition among China’s population and provides a valuable data point to facilitate scale-up efforts of China’s mental health system.

## Conclusion

These mental health literacy findings provide important new data to guide scale-up of China’s expansion from hospital-based delivery systems to a community-based mental health care system, as well as its shift from involuntary admissions of primarily psychotic disorders to voluntary treatment of a range of mental health problems. We confirmed an expected low overall level of accurate mental illness recognition nationally, with a particular gap between urban and rural respondents. These effects also vary for different age groups. In sum, this study captures baseline mental health literacy at a unique moment in China’s mental health reform history. Although the country has continued to make reforms since the time our study was conducted, these findings can still help shape China’s future development of mental health systems. In addition, the work may also benefit other lower middle income countries (LMIC) planning to undergo similar reforms to develop community mental health care delivery systems [38, 48]. Through a continued understanding of the processes that shape effective mental health treatment utilization, we intend our findings to promote the basic human right to mental health care and to alleviate the global burden of mental illness.

## Appendix 1

Vignette Examples for Men, Han Chinese Component of the Stigma in Global Context – Mental Health Study) SGC-MHS), 2011.*

### Major Depression

Zhang Dawei is a Han man. For the past two weeks Wei has been feeling really down. He wakes up in the morning with a flat heavy feeling that sticks with him all day long. He isn't enjoying things the way he normally would. In fact, nothing gives him pleasure. Even when good things happen, they don't seem to make Wei happy. He pushes on through his days, but it is really hard. The smallest tasks are difficult to accomplish. He finds it hard to concentrate on anything. He feels out of energy and out of steam. And even though Wei feels tired, when night comes he can't go to sleep. Wei feels pretty worthless, and very discouraged. Wei’s family has noticed that he hasn't been himself for about the last month and that he has pulled away from them. Wei just doesn't feel like talking.

### Schizophrenia

Zhang Dawei is a Han man. Up until a year ago, life was pretty okay for Wei. But then, things started to change. He thought that people around him were making disapproving comments, and talking behind his back. Wei was convinced that people were spying on him and that they could hear what he was thinking. Wei lost his drive to participate in his usual work and family activities and retreated to his home, eventually spending most of his day in his room. Wei was hearing voices even though no one else was around. These voices told him what do and what to think. He has been living this way for six months.

*****Referent for female is Zhang Xiao

## Abbreviations

APCMHD: Asia-Pacific Community Mental Health Development
DSM-IV: Diagnostic & statistical manual
LMIC: Low and middle-income countries
OLS: Ordinary least square
SGC-MHS: China from the stigma in global context – mental health study
